# Isolation and Identification of *Escherichia coli* O157:H7 Lytic Bacteriophage from Environment Sewage

**DOI:** 10.1155/2021/7383121

**Published:** 2021-08-11

**Authors:** Tessa Sjahriani, Eddy Bagus Wasito, Wiwiek Tyasningsih

**Affiliations:** ^1^Doctoral Program, Faculty of Medicine, Universitas Airlangga, Dr. Moestopo Road no. 47, Surabaya 60285, Indonesia; ^2^Department of Microbiology, Faculty of Medicine, Universitas Airlangga, Dr. Moestopo Road no. 47, Surabaya 60285, Indonesia; ^3^Department of Microbiology, Faculty of Veterinary Medicine, Universitas Airlangga C Campus, Mulyorejo Road, Surabaya 60115, Indonesia

## Abstract

*Escherichia coli* O157:H7 is one of the pathogenic bacteria causing foodborne disease. The use of lytic bacteriophages can be a good solution to overcome the disease. This study is aimed at isolating lytic bacteriophages from environmental sewage with *E. coli* O157:H7 bacterial cells. The sample used in this study was eight bacteriophages, and the technique used in identifying *E. coli* O157:H7 carriers of the *stx*1 and *stx*2 genes was PCR. The double layer plaque technique was used to classify bacteriophages. Plaque morphology, host specificity, and electron micrograph were used to identify the bacteriophages. The result obtained plaque morphology as a clear zone with the largest diameter size of 3.5 mm. Lytic bacteriophage could infect *E. coli* O157:H7 at the highest titer of 10 × 10^8^ PFU/mL. Bacteriophages have been identified as *Siphoviridae* and *Myoviridae*. Phage 3, phage 4, and phage 8 could infect *Atypical Diarrheagenic E. coli* 1 (*aDEC*1) due to their host specificity. The Friedman statistical tests indicate that lytic bacteriophage can significantly lyse *E. coli* O157:H7 (*p* = 0.012). The lysis of *E. coli* O157:H7 by phage 1, phage 2, phage 3, and phage 5 bacteriophages was statistically significant, according to Conover's posthoc test (*p* < 0.05). The conclusion obtained from this study is that lytic bacteriophages from environmental sewage could lyse *E. coli* O157:H7. Therefore, it could be an alternative biocontrol agent against *E. coli* O157:H7 that contaminates food causing foodborne disease.

## 1. Introduction

Foodborne diseases are diseases caused by consuming food and or drinks contaminated by various microorganisms or pathogenic microbes (foodborne pathogens) [1]. Foodborne diseases and cases of food poisoning are of wide concern in public health, especially in developing countries [2]. One of the key goals of national food safety programs is to reduce the number of cases of foodborne disease [3]. *Escherichia coli* O157:H7 is suspected of causing 63.000 instances of foodborne illness, 2.100 hospitalizations, 20 deaths, and a $ 271 million economic burden [4, 5]. *E. coli* O157:H7 was existed in the feces of asymptomatic children attending elementary school in Surabaya, East Java, Indonesia [6]. East Java is the largest contributor to the national cattle population, accounting for roughly 43% of the total, with beef production accounting for 20% of the total, or roughly 575,557 tonnes, and beef consumption accounting for 447,460 tonnes [7]. The Kalimas River, one of Surabaya's main waterways, has been found to be contaminated by market sewage from a nearby site that lacks a wastewater treatment plant [8–10]. Therefore, interventions with a focus on food safety are needed to prevent the occurrence of foodborne pathogens in both children and people in general [11].

*E. coli* O157:H7 could cause disease by toxin releasing called shiga-like toxin (*stx*), which could result in food poisoning [12]. Shiga toxin producing *E. coli* causes more than 2.5 million diseases worldwide each year, resulting 269 deaths [11]. Lytic bacteriophages provide a natural and nontoxic method to reduce and control the growth of human pathogenic bacteria because bacteriophages are part of the environmental ecosystem [13] and as a component of human microbiome [14]. Because antibiotics are known to cause resistance [15], this could be an alternative option. Herbal antibiotics, in addition to antibiotics, have a complex nature that is used to destroy bacteria and strengthen the immune system [16]. In this context, weak organic acids [primarily acetic acid, though work with citric acid has also been done on a small scale for many years to treat infections] have been used on a small scale to treat infections for many years; however, the majority of these publications are in the clinical literature and generally report single cases or small surveys [17]. Although many phytochemicals have antibacterial properties, they are not currently employed as commercial antibiotics. As a result, plants must be researched in order to have a proper understanding of their therapeutic properties, safety, and efficacy, so that herbal medicines can be utilized to replace and supplement synthetic pharmaceuticals [18].

Bacteriophages are mostly composed of nucleic acid, which is nontoxic. Therefore, it is beneficial as it has a low impact on the environment with relatively affordable costs. As a bacteriophage is used for a biocontrol agent, the interaction between bacteriophage proteins with biology or environmental components, animals, and the immune responses of a person which could be increased for potential negative effects needs to be considered (for example, when antibiotics make a deletion or elimination of the growth of bacteria, as a form of self defense, the bacteria will release a type of protein or toxin that can trigger a person's immune response) [19].

## 2. Materials and Methods

### 2.1. Bacterial Stock Culture Preparation

Stock culture of *E. coli* O157:H7 isolate stored in Tryptic Soy Broth Media (Oxoid, United Kingdom) with 15% glycerol at -20°C was obtained from the Microbiology Laboratory, Soetomo General Academic Hospital, Surabaya, East Java, Indonesia. Stock culture of *E. coli* O157:H7 was subcultured on Mac Conkey Agar plate media (Oxoid, UK), incubated at 37°C for 24 hours and stored at low temperature.

#### 2.1.1. Bacterial DNA Extraction

Bacterial DNA extraction referred to Geneaid (2017) procedural [20].

#### 2.1.2. Amplification of Bacterial DNA Target

PCR was performed using 2 mL of template in a 20 mL volume of the PCR PreMix (Promega corporation). The PCR mixture consisted of 1 U thermostable DNA polymerase, 250 mM dNTP, 50 mM Tris-HCl (pH 8.3), 40 mM KCl, and 1.5 mM MgCl_2_. PCR was carried out in a gene thermal cycler (Bio-Rad, Tokyo, Japan). The optimized cycle program of denaturation, annealing, and extension temperatures was as follows: 1 cycle of 2 min at 94°C; 35 cycles of 30 s at 94°C, 30 s at 55°C, and 1 min at 72°C; and 1 cycle of 5 min at 72°C.

Primer of shiga toxin genes of *stx*1 uses forward CAGTTAATGTGGTGGCGAAGG and reverse CACCAGACAATGTAACCGCTG of 1221 bp and *Stx*2 forward ATCCTATTCCCGGGAGTTACG-3 and reverse GCGTCATCGTATACACAGGAGC of 1247 bp [21]. The PCR products were analyzed using 2% agarose gel electrophoresis with ethidium bromide staining, with a 100 bp DNA Ladder Marker (Promega corporation). Electrophoresis was carried out at 100 volts for 35 minutes. Visualization of the band that appeared was done through a UV transilluminator and photographed [22] by Spectrolyne TC-312E/F, Japan.

### 2.2. Bacteriophage Preparation

The samples used for bacteriophage isolation were taken from traditional market sewage, abbatoir, and Kalimas River of Surabaya, Indonesia (as seen on Table 1). Each 15 mL of sample was taken using a sterile tube.

#### 2.2.1. Isolation of Bacteriophage

Sample filtration used the modified Thung et al. method, as much as 1 mL of liquid waste sample was diluted into 9 mL of Nutrient Broth (Oxoid, UK) media, centrifuged at 3000 rpm for 20 minutes, and then the supernatant was filtered using 0.45 *μ*m millipore membrane (Minisart, Sartorius). The 4.5 mL filtrate was then mixed with 0.5 mL of *E. coli* O157:H7 at exponential phase culture (McFarland 0.5 ~ 1.5 × 10^8^ CFU/mL) and added 5 mL of Nutrient Broth. Next, the mixture was incubated for 24 hours in a waterbath at 37°C. It was then centrifuged at 3000 rpm at 4°C for 15 minutes. The supernatant was taken by a syringe and filtered using 0.22 *μ*m millipore membrane (Minisart, Sartorius). The supernatant that has been filtered was inserted into a sterile tube and stored at low temperature [23].

#### 2.2.2. Lytic Bacteriophage Morphology by the Double Layer Agar Method

Bacteriophage stocks were then inoculated using the modified method of Bonilla et al. by the double layer plaque technique. A total of 100 *μ*L of lytic bacteriophage stock were diluted into the Ringer buffer with serial 10-fold dilutions. Then, each serial dilution of lytic bacteriophages was taken as much as 100 *μ*L and each was mixed with 100 *μ*L of *E. coli* O157:H7 bacteria at exponential phase culture (McFarland 0.5) into a new sterile Eppendorp tube and incubated at 37°C for 30 minutes. Soft agar consisting 70% nutrient agar media that was previously made was then warmed at 56°C. After that, the previous mixture was added by pouring technique on nutrient agar media. Incubation was carried out at 37°C for 24 hours [24], and then the plaque formed was observed as lytic bacteriophage existence [23].

As lytic bacteriophages were observed, the purification of lytic bacteriophages was carried out by the plaque formed using Pasteur pipettes and enriched, so that more plaques would produce. Each plaque was transferred to 10 mL of *E. coli* O157:H7 bacterial culture at exponential phase (McFarland 0.5) and incubated for 24 hours, then centrifuged at 3000 rpm at 4°C for 20 minutes. The bacteriophage filtrate was then filtered using 0.22 *μ*m millipore membrane (Minisart, Sartorius).

The result was in the form of a bacteriophage filtrate, and then it was carried out in an NA plate media. The plaque formed was then removed and inserted into the Ringer buffer. The bacteriophage suspension was vortexed and left about 5 to 10 minutes at room temperature, and so then the bacteriophages could attach the *E. coli* O157:H7 bacteria. Then, centrifugation was done at 3000 rpm at 4°C for 20 minutes for two replication times. The supernatant was filtered using a 0.22 *μ*m millipore filter membrane (Minisart, Sartorius) and then stored as bacteriophage stock at low temperature (4°C) [23].

#### 2.2.3. Bacteriophage Purification

A modified method of Thung et al. of bacteriophage propagation was used. The plaque formed previously was then removed and inserted into the Ringer buffer (4 : 1). The bacteriophage suspension was vortexed and left about 5 to 10 minutes at room temperature, and so then the bacteriophages could attach the *E. coli* O157:H7 bacteria. Then, centrifugation was done at 3000 rpm at 4°C for 20 minutes for two replication times. The supernatant was filtered using a 0.22 *μ*m millipore filter membrane (Minisart, Sartorius) and then stored as bacteriophage stock at low temperature (4°C) [23].

#### 2.2.4. Bacteriophage Propagation

Bacteriophage propagation was using the modified method of Bonilla et al., and a total of 10 mL of *E. coli* O157:H7 bacteria cultured in Nutrient Broth medium (Oxoid, UK) at exponential phase (McFarland 0.5) were centrifuged at 3000 rpm at 4°C for 20 minutes. The pellets formed were each infected with 100 *μ*L of lytic bacteriophages. Each mixture was incubated at 37°C for 30 minutes, and the mixture was then added to 10 mL of Nutrient Broth medium and incubated for 24 hours at 37°C. Then, each of them was centrifuged at 3000 rpm at 4°C for 20 minutes. The supernatant formed was taken with a syringe and filtered with a 0.22 *μ*m filter membrane (Minisart, Sartorius). Each supernatant that had been filtered was inserted into a sterile tube and stored as a bacteriophage stock at low temperature (4°C) [24] at Department of Microbiology, Faculty of Medicine, Universitas Airlangga, Surabaya, East Java, Indonesia.

#### 2.2.5. Bacteriophage Quantification

Bacteriophage quantification was measured by counting the amount of plaque formed in NA media plates as plaque forming units/mL (PFU/mL). Each lytic bacteriophage stock was diluted by 10-fold serial, and then 100 *μ*L from each dilution of the bacteriophage isolate was taken and transferred to 100 *μ*L of the *E. coli* O157:H7 bacterial culture, after incubated for 24 hours on Nutrient Broth medium (Oxoid, UK). The suspension was incubated for 30 minutes at 37°C. A total of 7 mL of soft agar that was previously made at 56°C were mixed. After that, each one of the suspensions was poured onto NA plate media (Oxoid, UK) and incubated at 37°C for 24 hours. After incubation, we then observed the plaque formed and expressed as PFU/mL [24].

### 2.3. Electron Micrograph of Bacteriophage

The Eijkman Institute in Jakarta, Indonesia, used transmission electron microscopy to identify the shape of the bacteriophage. A total of 10 *μ*L of bacteriophage were dropped onto a 400 mesh grid and left for 30 seconds. On carbon-coated grids, bacteriophage samples were negatively stained with 5 *μ*L of 2% (w/v) uranyl acetate. The grids were viewed using a JEM-1010 TEM (JEOL, Tokyo, Japan) [25].

### 2.4. Lysis of *E. coli* O157:H7 Bacteriophage

Lysis of *E. coli* O157:H7 by bacteriophages was using the modified method of Mirzaei and Nilsson and Aryal. One milliliter of *E. coli* O157:H7 bacteria that had been grown on MacConkey Agar plates was transferred onto Nutrient Broth media (Oxoid, UK) at exponential phase (McFarland 0.5), and each was infected with 1 mL of bacteriophage stock. Each mixture was then incubated for 2 hours, 4 hours, 6 hours, and 8 hours. The opacity assessment of McFarland densitometry was carried out to calculate the number of live bacteria [25, 26].

### 2.5. Bacterial Suspension Preparation for Host Specificity

The bacteria used for the host specificity test of lytic bacteriophages were *Atypical Diarrheagenic E. coli* isolates (a*DEC*1, a*DEC*2, a*DEC*3, a*DEC*4, a*DEC*5) (from hospital inpatient care of diarrhea) and keep storage clinical storage *Salmonella paratyphi A, Salmonella paratyphi B*, and *Shigella flexneri* isolates (from the Microbiology Laboratory of Medical Faculty of Universitas Airlangga Culture Collection, Surabaya, Indonesia). *Atypical Diarrheagenic E. coli* (a*DEC*1, a*DEC*2, a*DEC*3, a*DEC*4, a*DEC*5)*, S. paratyphi A*, *S. paratyphi B,* and *S. flexneri* isolates were stored in Tryptic Soy Broth Media with 15% glycerol at -20°C.

**Figure 1:**
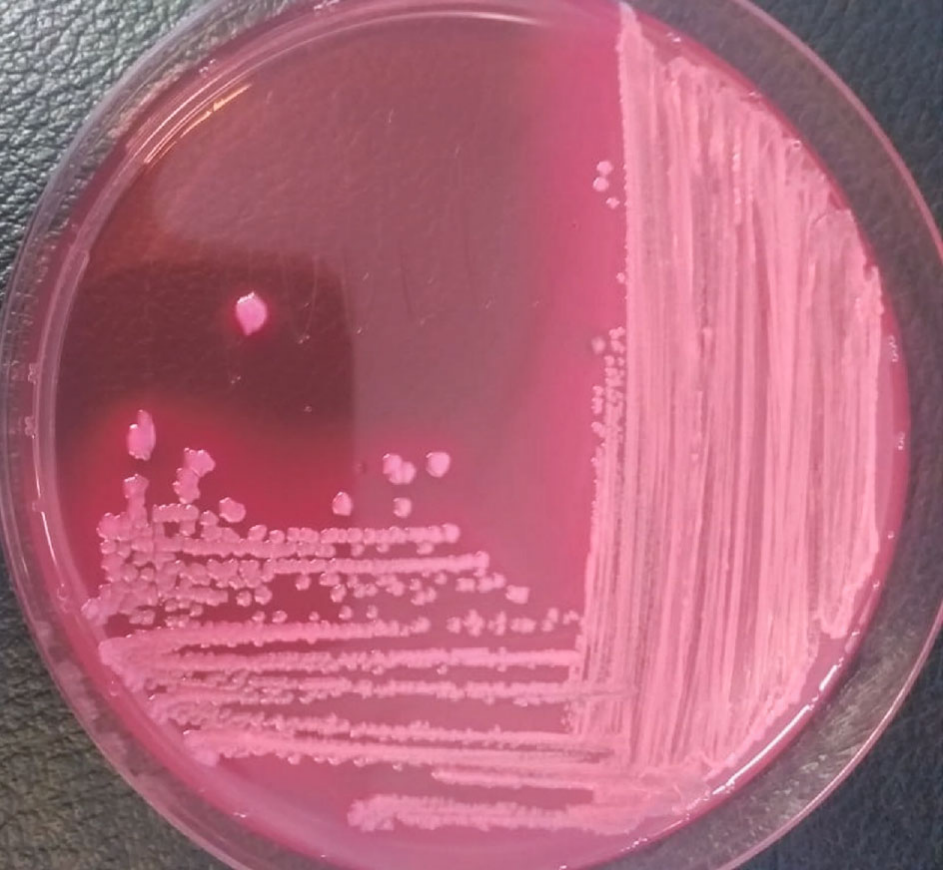
*Atypical Diarrheagenic E. coli* on MacConkey Agar.

**Figure 2:**
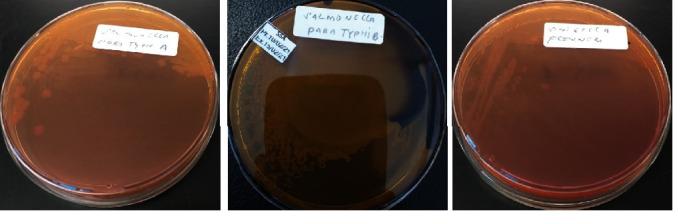
*S. paratyphi A, S. paratyphi B*, and *S. flexneri* on Salmonella Shigella Agar.

Stock culture of a*DEC*1, a*DEC*2, a*DEC*3, a*DEC*4, and a*DEC*5 was inoculated into Mac Conkey Agar plate media (Oxoid, UK) and incubated at 37°C for 24 hours. A positive result gives rise to a pink to rose red colonies, such as its ability to ferment lactose [27, 28] (Figure 1). Stock cultures of *S. paratyphi A*, *S. paratyphi B,* and *S. flexneri* isolates were inoculated into Salmonella Shigella Agar plate media (Oxoid, UK) and incubated at 37°C for 24 hours. A positive result gave rise to colorless for *S. paratyphi A* and *S. flexneri,* shown as colorless with black centers for *S. paratyphi B* as its ability to produce hydrogen sulfide (H_2_S) [29] (Figure 2).

*Atypical Diarrheagenic E. coli, S. paratyphi A*, *S. paratyphi B,* and *S. flexneri* cultures were then identified by biochemical assay on Triple Sugar Iron Agar/TSIA (Oxoid, UK), Sulfate Indol Motility/SIM (Oxoid, UK), Simmons Citrate Agar/SCA (Oxoid, UK), and Urea broth (Oxoid, UK) [30] (see Table 2).

### 2.6. Host Specificity of Bacteriophages

In cultures of *aDEC1, aDEC2, aDEC3, aDEC4, aDEC5, S. paratyphi A, S. paratyphi B,* and *S. flexneri*, bacteriophage host specificity was observed using 100 *μ*L of each bacterium previously grown in Tryptic Soy Broth Media (Oxoid, UK) by exponential phase (McFarland 0.5), mixed with 100 *μ*L of lytic bacteriophages stock, and diluted into the Ringer buffer with serial 10-fold dilutions. Then, each serial dilution of lytic bacteriophage was taken as much as 100 *μ*L into a new sterile Eppendorf tube and incubated at 37°C for 30 minutes for the lytic bacteriophages to attach. A total of 7 mL of soft agar that was previously made at 56°C was mixed by pouring technique on Nutrient Agar plate media, and incubation was carried out at 37°C for 24 hours. Then, the plaque formed was observed and counted [23].

### 2.7. Statistical Analysis

Statistical analysis used in this study was a nonparametric test in the form of the Friedman test. A significant result was continued with Conover's posthoc.

## 3. Results and Discussion

### 3.1. Detection of Shiga Toxin Genes

**Figure 3:**
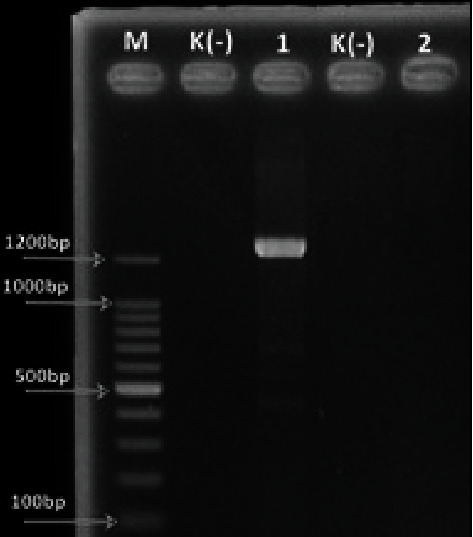
Detection of the shiga toxin gene of *E. coli* O157:H7 (M: marker; K: control; 1: *stx*1; 2: *stx*2).

The results of molecular identification of *E. coli* O157:H7 isolates carrying the *stx*1 and *stx*2 genes using the PCR technique show that only the *stx*1 gene was borne by *E. coli* O157:H7 isolates, as shown in Figure 3:

In contrast to the *stx*2 gene, this study discovered the *stx*1 gene formed by *E. coli* O157:H7 to the present in a 1220 bp band approximately. This gene was also found in the feces of children in Southern Iran [31] and human feces with clinical manifestations in Japan [32]. As a result, *E. coli* O157:H7 must also be monitored as a foodborne pathogen.

### 3.2. Bacteriophage Sample

The samples were collected from the environmental sources as shown in the following table:

### 3.3. Isolation of Bacteriophage

**Figure 4:**
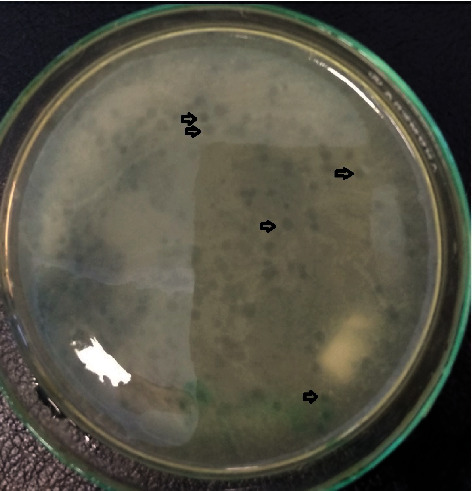
Lytic bacteriophage morphology by the double layer plaque technique.

Plaques in *E. coli* O157:H7 carrying shiga toxin gene cultures grown in the double layer plaque indicate the presence of lytic bacteriophages (see Figure 4).

Bacteriophages could affect bacterial lysis because they are able to recognize receptors on the surface of the host bacteria, so that bacteriophages are able to transfer their genetic material into the host cell and replicate in the host cell causing lysis [33]. From Table 3, it can be seen that the diameter size was around 0.7 to 3.5 mm. Topka et al. obtained bacteriophages with clear colors with diameters of around 2-3 mm [34]. Regarding this, Yazdi et al. conducted a study with diameters of around 1.5-2 mm [35].

There are several factors that could affect plaque formation. Rapidity to lyse bacterial cells was one factor that could determine plaque morphology and size. This factor could cause different appearances of plaques, either in morphology or in size [36]. Plaque diameter was closely related to propagation, and having the right size was desirable for an effective lytic bacteriophage [37].

The rate of plaque formation was influenced by environmental conditions (temperature, pH, and aeration) and the accessibility of the bacteriophage to the target bacteria [38, 39]. Previous studies also reported that cofactors such as Ca^2+^ ions can stabilize the fragile interface of the virion with its receptors [40, 41]. The size and amount of bacteriophage plaque resulting from host cell infection are related to the ability of the bacteriophage to replicate in the host cell. Plaque size is also influenced by several factors, such as agar concentration, incubation conditions, and the log phase of host bacteria [42].

### 3.4. Concentration of Lytic Bacteriophage

The concentrations of the purified bacteriophages were then calculated based on the amount of plaque formed. The amount of plaque formed was then calculated in plaque forming units (PFU/mL), which is a measure of the amount of virus infective per volume of fluid. In addition, the lytic bacteriophage concentration was calculated as seen in Table 4:

Bacteriophage quantification was measured by counting the amount of plaque formed, and the concentration obtained was of 5 × 10^3^ to 10 × 10^8^ PFU/mL. The number of bacteriophages previously detected was >10^8^ PFU/mL of *Bacillus cereus* bacteriophage [33], 10^15^ PFU/mL of *Klebsiella pneumoniae* bacteriophage [43], 5 × 10^12^ PFU/mL of *E. coli* bacteriophage [44], 1.2 × 10^16^ PFU/mL [45], and 5 × 10^6^ PFU/mL for T4 bacteriophage [46], and 1.3 × 10^11^ PFU/mL [23], 2.1 × 10^10^ PFU/mL [34], and 2.62 × 10^10^ PFU/mL of *E. coli* bacteriophage [33]. Marti et al. conducted a study with *Salmonella* spp. bacteriophages with titers of 5 × 10^8^ PFU/mL [47]. Bao et al. obtained titers of 5 × 10^7^ PFU/mL [48].

Other studies have shown similar results with bacteriophage titres between 10^8^ and 10^11^ PFU/mL, this could be due to the optimum plating condition, and other parameters such as the buffer in which the bacteriophages were suspended, the incubation media [49], Ca^2+^ ions which can stabilize the fragile interface of the virion with its receptors [40, 41], and the different bacteriophage environment surroundings could also be the influence [23, 50]. This study used Ringer buffer consisting of 1.55 g C_3_H_5_NaO_3_, 3 g NaCl, 0.15 g KCl, and 0.1 g CaCl_2_.2H2O, while other studies used SM buffer consisting of 5.8 g NaCl, 2 g MgSO_4_·7H2O, 50 mM Tris-Cl (pH 7.5), and 5 mL gelatine in 1 L H_2_O [23, 51, 52].

### 3.5. Electron Micrograph of Bacteriophage

**Figure 5:**
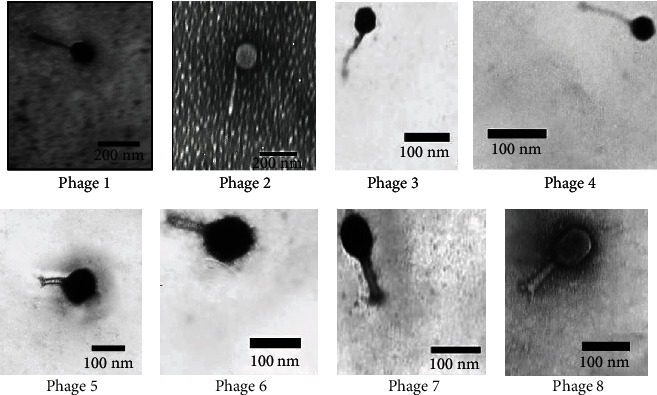
Electron micrograph of negatively stained bacteriophage.

The morphology of bacteriophages, such as the head and tail shapes, was discovered to be identical to the order of Caudovirales [53, 54], based on observations (see Figure 5).

Bacteriophages have a head and tail that vary in size, as seen in Table 5:

Based on the anatomy of the tail, Caudovirales was split into three groups. *Myoviridae* were bacteriophages with a long contractile tail, while *Siphoviridae* had a long noncontractile tail [54]. The head diameter of *Siphoviridae* bacteriophage ranged from 50 to 133.3 nm, while *Myoviridae* bacteriophage had a head diameter of 83.3 to 100 nm. The tail length of *Siphoviridae* bacteriophages ranged from 100 to 288 nm, while the tail length of *Myoviridae* bacteriophages ranged from 83.3 to 100 nm. The tail diameter of *Siphoviridae* bacteriophages ranged from 6.3 to 17.4 nm, while the tail length of *Myoviridae* bacteriophages ranged from 10 to 16.7 nm (see Table 5).

Bao et al. found the bacteriophage *Siphoviridae* with a tail length of 103.57 nm and a head diameter of 57.14 nm and *Myoviridae* bacteriophage with a head diameter of 74.3 nm and a tail length of 114.2 nm from chicken waste in chicken farms in China [48], while Lukman et al. also found EPEC bacteriophages with a head diameter of 67-70 nm and a tail length of 83-90 nm as *Myoviridae* from chickens and beef offal from traditional markets in Tangerang, Indonesia [33]. To ensure bacteriophage classification, more study, such as bacteriophage genome sequencing, is required.

### 3.6. Lysis of *E. coli* O157:H7 by Bacteriophage

One mL of *E. coli* O157:H7 was injected with 1 mL of bacteriophage stock into Triptic Soy Broth media (Oxoid, UK) at 37°C at an incubation interval of 0 hour, 2 hours, 4 hours, 6 hours, and 8 hours. The lysis of *E. coli* O157:H7 is as seen on Table 6:

The ability of LBP to lyse *E. coli* O157:H7 can be seen. In comparison to the amount of *E. coli* O157:H7 in the control, which was 1.5 × 10^8^ CFU/mL without lytic bacteriophage injection, the lowest value of *E. coli* O157; H7 obtained was 3.7 × 10^7^ CFU/mL, with a maximum of 1.2 × 10^8^ CFU/mL (phage 1). The following table shows the reduction in *E. coli* O157:H7 caused by lytic bacteriophage:

After the 8-hour incubation period, lytic bacteriophage was able to lyse *E. coli* O157:H7 by 75% (see Table 7). Yazdi et al. stated that higher bacteriophage concentrations resulted in a faster reduction in the bacterial count, which could be due to an increased attachment rate at higher bacteriophage titers [41]. The speed of plaque formation is considered related to the multiplication rate of bacteriophages. The more bacteriophages are produced, and the more bacterial cells are lysed [38, 42]. In addition, the ability of lysis was also influenced by the multiplicity of infection (MOI), which is the ratio of bacteriophages to the number of target bacteria [53]. Bacterial growth will increase along with the decrease in MOI [33], but MOI cannot completely inhibit cell growth [55].

At first glance, the *Myoviridae* bacteriophage was faster than the *Siphoviridae* bacteriophage in lysing *E. coli* O157:H7 at the beginning of the infection time, but it was not seen at subsequent incubation times and obtained the same number of lysis as the *Siphoviridae* bacteriophage at the end of the incubation period (after 8 hours). Based on these characteristics, more research is needed on the lysis of bacteriophages with a higher number of *Siphoviridae* and *Myoviridae* bacteriophages, as well as a bigger number of *E. coli* O157:H7 bacterium.

### 3.7. Host Specificity of Bacteriophages

**Figure 6:**
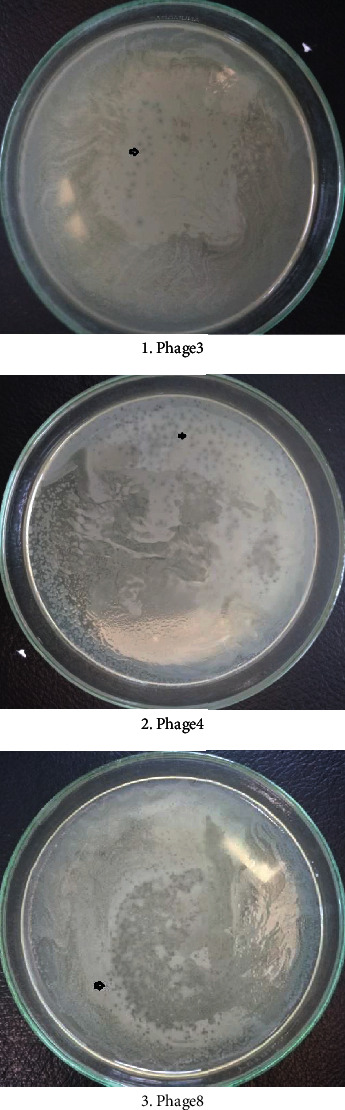
Lytic bacteriophage 3, 4, and 8 plaque formation on *Atypical Diarrheagenic E. coli* 1.

The host's specificity is depicted in Figure 6 as plaque formation:

Phage 3, phage 4, and phage 8 were found to have plaque formation in *aDEC*1 (see Figure 6), with *aDEC*2, *aDEC*3, *aDEC*4, *aDEC*5, *S. paratyphi* A, *S. paratyphi* B, and *S. flexneri*, and lytic bacteriophage plaque development was not seen. In contrast to *aDEC*2, *aDEC*3, *aDEC*4, *aDEC*5, *S. paratyphi A*, *S. paratyphi B*, and *S. flexneri*, the presence of plaque in host specificity testing with *aDEC*1 indicates that phage 3, phage 4, and phage 8 infect other serotypes in one bacterial species, implying that the surface of *aDEC*1 bacterial cells has the same particular receptors against phage 3, phage 4, and phage 8. In this regard, Akhtar et al. discovered that *Salmonella enterica* lytic bacteriophages have a host specificity for *S. typhimurium* [56].

Jamal et al.'s study revealed that bacteriophages that lyse *K. pneumoniae* could not infect other bacteria, which suggest a narrow host range among other different bacterial strains [40]. It is similar to Abatangelo et al. who studied *Staphylococcus aureus* bacteriophages [57]. Bao et al. and Jurczak et al. found that *Salmonella enterica* bacteriophages could also lyse *E. coli* [48, 58]. Chen et al. found bacteriophages which lysed *Pasteurella multocida* A, and *P* capsular type was not able to lyse strains with D or F capsular type or other Gram-negative bacteria, including *E. coli, Salmonella* spp., and *Bordetella bronchiseptica* [59]. Lukman et al. also found a bacteriophage that can lyse EPEC and EHEC [33]. Further research on bacteriophage host specificity on another pathogenic *E. coli* is required based on these traits.

The narrow host specificity of bacteriophages may be viewed as a disadvantage. This characteristic restricts the number of bacteria where the selection for bacteriophage resistance mechanisms can occur in comparison to chemical antibiotics. This perhaps could be circumvented as cocktail bacteriophages with the aim of being able to lyse a wider range of bacterial species. Otherwise, it can be an advantage as it offers fewer side effects on natural flora. Therefore, the subject will not be susceptible to superinfection [19, 60].

### 3.8. Statistical Analysis

Statistical analysis using JASP 0.14.1.0 programme described a nonparametric test. The Friedman test conducted a significant effect in the lyse of *E. coli* O157:H7 by lytic bacteriophages (*p* = 0.012), with estimating a large effect size of Kendall's W by 0.993 [61] (see Table 8):

From Conover's posthoc test, the *p* value was obtained for the effect of all groups as written in the following table:

Phage 1 had a statistically significant difference in *E. coli* O157:H7 quantity compared to phage 6 (*p* = 0.022), and LBP7 (*p* = 0.022), whereas phage 2 had a statistically significant difference in *E. coli* O157:H7 amount compared to phage 3 (*p* = 0.036), phage 6 (*p* = 0.014), and phage 7 (*p* = 0.014), according to Conover's posthoc test. In addition, phage 5 had a significantly different impact in the lyse of *E. coli* O157:H7 relative to phage 6 (*p* = 0.036) and phage 7 (*p* = 0.036). Phage 6 had a significantly different effect in the lyse of *E. coli* O157:H7 compared to phage 8 (*p* = 0.057), and phage 7 had a significantly different effect in the lyse of *E. coli* O157:H7 compared to phage 8 (*p* = 0.057) (see Table 9).

On phage 6 and phage 7, the amount of *E. coli* O157:H7 was shown to be lower than on phage 1, the number of *E. coli* O157:H7 detected by phage 3, phage 6, and phage 7 was statistically lower than phage 2, and the number of *E. coli* O157:H7 detected by phage 6 and phage 7 produced a lower number of *E. coli* O157:H7 than phage 5.

## 4. Conclusions

Lytic bacteriophage identified from environmental sewage in Surabaya, Indonesia, shows plaque morphology as a clear zone with the largest diameter size of 3.5 mm, and lytic bacteriophage could infect *E. coli* O157:H7 carrying the *stx*1 gene at the highest titer of 10 × 10^8^ PFU/mL. Bacteriophages have been identified as *Siphoviridae* and *Myoviridae* by electron micrograph.

It was studied that phage 3, phage 4, and phage 8 could infect *Atypical Diarrheagenic E. coli* 1 (*aDEC*1) due to their host specificity. The Friedman statistical tests indicate that lytic bacteriophage can significantly lyse *E. coli* O157:H7 (*p* = 0.012). The lysis of *E. coli* O157:H7 by phage 1, phage 2, phage 3, and phage 5 bacteriophages was statistically significant, according to Conover's posthoc test (*p* < 0.05). The conclusion obtained from this study is that lytic bacteriophages from environmental sewage could lyse *E. coli* O157:H7. Therefore, it could be an alternative biocontrol agent against *E. coli* O157:H7 that contaminates food causing foodborne disease.

## Figures and Tables

**Table 1:** Collection of bacteriophage samples.

No	Bacteriophage samples	Longitude	Latitude	Date
1	Cow sewage from abbatoir	7°13′13.2^″^S	112°44′44.3^″^E	August 31, 2020
2	Goat sewage from abbatoir	7°13′13.2^″^S	112°44′44.3^″^E	August 31, 2020
3	Pig sewage from abbatoir	7°13′13.2^″^S	112°44′44.3^″^E	August 31, 2020
4	Abbatoir drainage sewage	7°13′13.2^″^S	112°44′44.3^″^E	August 31, 2020
5	Chicken sewage from traditional market	7°16′39.5^″^S	112°44′36.4^″^E	August 31, 2020
6	Cow sewage from traditional market	7°18′06.6^″^S	112°44′13.6^″^E	August 31, 2020
7	Fish sewage from traditional market	7°16′28.2^″^S	112°44′35.4^″^E	August 31, 2020
8	Kalimas river sewage	7°16′36.0^″^S	112°44′37.6^″^E	August 31, 2020

**Table 2:** Biochemical assay on *Atypical Diarrheagenic E. coli*, *S. paratyphi A*, *S. paratyphi B*, and *S. flexneri.*

	*aDEC*	*S. Paratyphi* A	*S. Paratyphi* B	*S. Flexneri*
	(*n* = 5)	(*n* = 1)	(*n* = 1)	(*n* = 1)
TSIA	A/A, H_2_S-, gas+	A/K, S-, gas+	A/K, S+, gas +	A/K, S-, gas-
SIM	-, +, +	-, -, +	S,+	-, -, -
SCA	—	+	—	—
Urease	—	—	—	—

Note: *aDEC*: *Atypical Diarrheagenic E. coli*; A : acid; K: alkaline.

**Table 3:** Plaque diameter size of lytic bacteriophage.

Sample	Plaque diameter (mm)	Turbidity
Phage 1	2	Clear
Phage 2	2	Clear
Phage 3	3.5	Clear
Phage 4	2	Clear
Phage 5	1.5	Clear
Phage 6	2	Clear
Phage 7	0.7	Clear
Phage 8	1	Clear

**Table 4:** The concentration of lytic bacteriophage.

Sample	PFU/mL
Phage 1	9.2 × 10^7^
Phage 2	3.4 × 10^4^
Phage 3	3.0 × 10^8^
Phage 4	10.0 × 10^8^
Phage 5	3.9 × 10^6^
Phage 6	4.3 × 10^6^
Phage 7	5.4 × 10^3^
Phage 8	3.0 × 10^7^

**Table 5:** Bacteriophage: morphology of electron micrographs.

Bacteriophage	Family	Head diameter (nm)	Tail length (nm)	Tail diameter (nm)
Phage 1	*Siphoviridae*	113	200	17.4
Phage 2	*Siphoviridae*	133.3	288	10.3
Phage 3	*Siphoviridae*	50	100	8.7
Phage 4	*Siphoviridae*	50	100	6.3
Phage 5	*Myoviridae*	100	100	10
Phage 6	*Myoviridae*	100	71.4	14.3
Phage 7	*Myoviridae*	100	100	13.3
Phage 8	*Myoviridae*	83.3	83.3	16.7

**Table 6:** The number of *E. coli* O157:H7 by lytic bacteriophage.

	Phage 1	Phage 2	Phage 3	Phage 4	Phage 5	Phage 6	Phage 7	Phage 8
	*Siphoviridae*				*Myoviridae*			
Control	1.5 × 10^8^	1.5 × 10^8^	1.5 × 10^8^	1.5 × 10^8^	1.5 × 10^8^	1.5 × 10^8^	1.5 × 10^8^	1.5 × 10^8^
With phage	1.2 × 10^8^	1.1 × 10^8^	7.0 × 10^7^	7.0 × 10^7^	6.7 × 10^7^	6.1 × 10^7^	6.4 × 10^7^	6.4 × 10^7^
2 hours	5.8 × 10^7^	5.8 × 10^7^	5.2 × 10^7^	5.5 × 10^7^	5.8 × 10^7^	5.2 × 10^7^	4.3 × 10^7^	5.8 × 10^7^
4 hours	4.3 × 10^7^	5.5 × 10^7^	3.9 × 10^7^	4.3 × 10^7^	4.6 × 10^7^	4.0 × 10^7^	4.0 × 10^7^	5.5 × 10^7^
6 hours	3.7 × 10^7^	4.0 × 10^7^	3.7 × 10^7^	3.7 × 10^7^	4.3 × 10^7^	3.7 × 10^7^	3.7 × 10^7^	4.0 × 10^7^
8 hours	3.7 × 10^7^	3.7 × 10^7^	3.7 × 10^7^	3.7 × 10^7^	3.7 × 10^7^	3.7 × 10^7^	3.7 × 10^7^	3.7 × 10^7^

Control: *E. coli* O157:H7 without bacteriophage.

**Table 7:** Reduction of *E. coli* O157:H7 by lytic bacteriophage.

	Phage 1	Phage 2	Phage 3	Phage 4	Phage 5	Phage 6	Phage 7	Phage 8
	*Siphoviridae*				*Myoviridae*			
0 hour	20%	27%	53%	53%	55%	59%	57%	57%
2 hours	61%	61%	65%	63%	61%	65%	71%	61%
4 hours	71%	63%	74%	71%	69%	73%	73%	63%
6 hours	75%	73%	75%	75%	71%	75%	75%	73%
8 hours	75%	75%	75%	75%	75%	75%	75%	75%

**Table 8:** Friedman test of *E. coli* O157:H7 by bacteriophage.

Factor	*p*	Kendall's *W*
Lysis of *E. coli* O157; H7 by bacteriophage	0.012	0.993

**Table 9:** Conover's posthoc comparisons.

		*W*_*i*_	*W*_*j*_	*p*
Phage 1	Phage 2	34.500	36.000	0.834
	Phage 3	34.500	20.500	0.057
	Phage 4	34.500	25.500	0.214
	Phage 5	34.500	33.000	0.834
	Phage 6	34.500	17.500	0.022
	Phage 7	34.500	17.500	0.022
	Phage 8	34.500	31.500	0.676

Phage 2	Phage 3	36.000	20.500	0.036
	Phage 4	36.000	25.500	0.149
	Phage 5	36.000	33.000	0.676
	Phage 6	36.000	17.500	0.014
	Phage 7	36.000	17.500	0.014
	Phage 8	36.000	31.500	0.531

Phage 3	Phage 4	20.500	25.500	0.487
	Phage 5	20.500	33.000	0.088
	Phage 6	20.500	17.500	0.676
	Phage 7	20.500	17.500	0.676
	Phage 8	20.500	31.500	0.131

Phage 4	Phage 5	25.500	33.000	0.299
	Phage 6	25.500	17.500	0.269
	Phage 7	25.500	17.500	0.269
	Phage 8	25.500	31.500	0.405

Phage 5	Phage 6	33.000	17.500	0.036
	Phage 7	33.000	17.500	0.036
	Phage 8	33.000	31.500	0.834

Phage 6	Phage 7	17.500	17.500	1.000
	Phage 8	17.500	31.500	0.057

Phage 7	Phage 8	17.500	31.500	0.057

## Data Availability

The data used to support the findings of this study are included within the article.

## References

[1] Junillon T., Vimont A., Mosticone D. (2012). Simplified detection of food-borne pathogens: an *in situ* high affinity capture and staining concept. *Journal of Microbiological Methods*.

[2] Carbas B., Cardoso L., Coelho A. C. (2013). Investigation on the knowledge associated with foodborne diseases in consumers of northeastern Portugal. *Food Control*.

[3] Azevedo I., Albano H., Silva J., Teixeira P. (2014). Food safety in the domestic environment. *Food Control*.

[4] Hoffmann S., Maculloch B., Batz M. (2015). Economic burden of major foodborne illnesses acquired in the United States. https://www.ers.usda.gov/webdocs/publications/43984/52807_eib140.pdf?v=2385.8.

[5] Poxleitner M., Pope W., Sera D. J., Sivanathan V. H. G. (2016). *Phage discovery Guide*.

[6] Syahrul F., Wahyuni C. U., Notobroto H. B., Wasito E. B., Adi A. C., Dwirahmadi F. (2020). Transmission media of foodborne diseases as an index prediction of diarrheagenic Escherichia coli: study at elementary school, Surabaya, Indonesia. *International Journal of Environmental Research and Public Health*.

[7] Bisnis.com (2019). East Java cattle population targeted to grow 3.5% this year. https://surabaya.bisnis.com/read/20190709/532/1121842/populasi-sapi-jatim-ditarget-tumbuh-35-tahun-ini.

[8] Detiknews (2018). Keputran poultry market that smells badly unpacked. https://news.detik.com/berita-jawa-timur/d-4198620/lapak-pasar-unggas-keputran-yang-berbau-tak-sedap-dibongkar.

[9] (2019). Surabaya City Government evicts 40 poultry stands at Keputran market. https://jatimnet.com/pemkot-surabaya-gusur-40-stand-unggas-di-pasar-keputran.

[10] (2019). Smells, PD pasar regulates waste disposal of Keputran poultry traders. https://jatimnet.com/berbau-pd-pasar-atur-pembuangan-limbah-pedagang-unggas-keputran.

[11] Kirk M. D., Pires S. M., Black R. E. (2015). World Health Organization estimates of the global and regional disease burden of 22 foodborne bacterial, protozoal, and viral diseases, 2010: a data synthesis. *PLoS Medicine*.

[12] Fathi J., Ebrahimi F., Nazarian S., Tarverdizade Y. (2017). Purification of shiga-like toxin from Escherichia coli O157: H7 by a simple method. *Journal of Applied Biotechnology Reports*.

[13] Bhardwaj N., Bhardwa S. K., Deep A., Dahiya S., Kapoor S. (2015). Lytic bacteriophages as biocontrol agents of foodborne pathogens. *Asian Journal of Animal and Veterinary*.

[14] Huh H., Wong S., St Jean J., Slavcev R. (2019). Bacteriophage interactions with mammalian tissue: therapeutic applications. *Advanced Drug Delivery Reviews*.

[15] Aslam B., Wang W., Arshad M. I. (2018). Antibiotic resistance: a rundown of a global crisis. *Infection and Drug Resistance*.

[16] Basappa K., Venu G. J. (2013). Natural alternatives to antibiotic agents. *Asian Journal of Biomedical and Pharmaceutical Sciences*.

[17] Bushell F. M. L., Tonner P. D., Jabbari S., Schmid A. K., Lund P. A. (2019). Synergistic impacts of organic acids and pH on growth of Pseudomonas aeruginosa: a comparison of parametric and Bayesian non-parametric methods to model growth. *Front Microbiol*.

[18] Mathur P. (2018). Need of herbal antibiotics. *Clinical Pathology & Research Journal*.

[19] Loc-Carrillo C., Abedon S. T. (2011). Pros and cons of phage therapy. *Bacteriophage*.

[20] Geneaid (2017). *Presto ™ Mini gDNA Bacteria Kit*.

[21] MOON G. S., KIM W. J., SHIN W. S. (2004). Optimization of rapid detection of Escherichia coli O157:H7 and listeria monocytogenes by PCR and application to field test. *Journal of Food Protection*.

[22] Taha K. M. (2016). Agarose gel electrophoresis agriculture college-animal resources preparation of agarose gel. https://www.researchgate.net/publication/308904762_Agarose_Gel_electrophoresis.

[23] Thung T. Y., Siti Norshafawatie B. M. F., Premarathne J. M. K. J. K. (2017). Isolation of food-borne pathogen bacteriophages from retail food and environmental sewage. *International Food Research Journal*.

[24] Bonilla N., Rojas M. I., Cruz G. N. F. (2016). Phage on tap-a quick and efficient protocol for the preparation of bacteriophage laboratory stocks. *PeerJ*.

[25] Mirzaei M. K., Nilsson A. S. (2015). Correction: Isolation of phages for phage therapy: a comparison of spot tests and efficiency of plating analyses for determination of host range and efficacy. *PLoS One*.

[26] Aryal S. (2020). McFarland standards principle preparation uses limitations.pdf. https://microbenotes.com/mcfarland-standards/.

[27] Dikobe B. T., Sithebe N. P., Ateba C. N. (2011). Detection ofE. coliisolates in water from Setumo Dam Mmabatho Area – North West Province, South Africa usingmdhspecific PCR analysis. *Journal of Human Ecology*.

[28] Aryal S. (2018). MacConkey Agar-composition, principle, uses, preparation and colony morphology. https://microbiologyinfo.com/macconkey-agar-composition-principle-uses-preparation-and-colony-morphology/.

[29] Aryal S. (2011). Salmonella Shigella (SS) Agar-composition, principle, uses, preparation and result interpretation. https://microbiologyinfo.com/salmonella-shigella-ss-agar-composition-principle-uses-preparation-and-result-interpretation/.

[30] Varghese N., Joy P. P., Varghese N. (2016). Microbiology laboratory manual. https://www.researchgate.net/publication/306018042_Microbiology_Laboratory_Manual#fullTextFileContent.

[31] Kargar M., Homayoon M. (2015). Prevalence of shiga toxins (*stx*_1_, *stx*_2_), *eaeA* and *hly* genes of *Escherichia coli* O157:H7 strains among children with acute gastroenteritis in southern of Iran. *Asian Pacific Journal of Tropical Medicine*.

[32] Hoang Minh S., Kimura E., Hoang Minh D., Honjoh K., Miyamoto T. (2015). Virulence characteristics of shiga toxin-producing Escherichia coli from raw meats and clinical samples. *Microbiology and Immunology*.

[33] Lukman C., Yonathan C., Magdalena S., Waturangi D. E. (2020). Isolation and characterization of pathogenic Escherichia coli bacteriophages from chicken and beef offal. *BMC Research Notes*.

[34] Topka G., Bloch S., Nejman-Faleńczyk B. (2019). Characterization of bacteriophage vB-EcoS-95, isolated from urban sewage and revealing extremely rapid lytic development. *Front Microbiol*.

[35] Yazdi M., Bouzari M., Ghaemi E. A., Shahin K. (2020). Isolation, characterization and genomic analysis of a novel bacteriophage VB_EcoS-Golestan infecting multidrug-resistant *Escherichia coli* isolated from urinary tract infection. *Scientific Reports*.

[36] Gallet R., Kannoly S., Wang I. N. (2011). Effects of bacteriophage traits on plaque formation. *BMC Microbiology*.

[37] Amarillas L., Rubí-Rangel L., Chaidez C., González-Robles A., Lightbourn-Rojas L., León-Félix J. (2017). Isolation and characterization of phiLLS, a novel phage with potential biocontrol agent against multidrug-resistant Escherichia coli. *Frontiers in Microbiology*.

[38] Ly-Chatain M. H. (2014). The factors affecting effectiveness of treatment in phages therapy. *Frontiers in Microbiology*.

[39] Fister S., Robben C., Witte A. K., Schoder D., Wagner M., Rossmanith P. (2016). Influence of environmental factors on phage-bacteria interaction and on the efficacy and infectivity of phage P100. *Frontiers in Microbiology*.

[40] Jamal M., Hussain T., Das C. R., Andleeb S. (2015). Characterization of siphoviridae phage Z and studying its efficacy against multidrug-resistant Klebsiella pneumoniae planktonic cells and biofilm. *Journal of Medical Microbiology*.

[41] Yazdi M., Bouzari M., Ghaemi E. A. (2018). Isolation and characterization of a lytic bacteriophage (vB_PmiS-TH) and its application in combination with ampicillin against planktonic and biofilm forms of *Proteus mirabilis* isolated from urinary tract infection. *Journal of Molecular Microbiology and Biotechnology*.

[42] Hyman P. (2019). Phages for phage therapy: Isolation, characterization, and host range breadth. *Pharmaceuticals*.

[43] Gupta R., Bekele W., Ghatak A. (2013). Harvesting energy of interaction between bacteria and bacteriophage in a membrane-less fuel cell. *Bioresource Technology*.

[44] Warner C. M., Barker N., Lee S. W., Perkins E. J. (2014). M13 bacteriophage production for large-scale applications. *Bioprocess and Biosystems Engineering*.

[45] Sochocka M., Tomczyk T., Sobczyński M., Szermer-Olearnik B., Boratyński J. (2015). The kinetics of Escherichia coli B growth and bacteriophage T4 multiplication in SM-1 novel minimal culture medium. *The Journal of General and Applied Microbiology*.

[46] Nabergoj D., Kuzmić N., Drakslar B., Podgornik A. (2018). Effect of dilution rate on productivity of continuous bacteriophage production in cellstat. *Applied Microbiology and Biotechnology*.

[47] Marti R., Zurfluh K., Hagens S., Pianezzi J., Klumpp J., Loessner M. J. (2013). Long tail fibres of the novel broad-host-range T-even bacteriophage S16 specifically recognizeSalmonellaOmpC. *Molecular Microbiology*.

[48] Bao H., Zhang H., Wang R. (2011). Isolation and characterization of bacteriophages of *Salmonella enterica* serovar Pullorum. *Poultry Science*.

[49] Cormier J., Janes M. (2014). A double layer plaque assay using spread plate technique for enumeration of bacteriophage MS2. *Journal of Virological Methods*.

[50] Denes T., Wiedmann M. (2014). Environmental responses and phage susceptibility in foodborne pathogens: implications for improving applications in food safety. *Current Opinion in Biotechnology*.

[51] Jakočiūnė D., Moodley A. (2018). A rapid bacteriophage dna extraction method. *Methods and Protocols*.

[52] Gutiérrez D., Fernández L., Rodríguez A., García P. (2018). Practical method for isolation of phage deletion mutants. *Methods and Protocols*.

[53] Chibani C. M., Farr A., Klama S., Dietrich S., Liesegang H. (2019). Classifying the unclassified: a phage classification method. *Viruses*.

[54] Ackermann H. W., Prangishvili D. (2012). Prokaryote viruses studied by electron microscopy. *Archives of Virology*.

[55] Tomat D., Migliore L., Aquili V., Quiberoni A., Balagué C. (2013). Phage biocontrol of enteropathogenic and shiga toxin-producing Escherichia coli in meat products. *Frontiers in Cellular and Infection Microbiology*.

[56] Akhtar M., Viazis S., Diez-Gonzalez F. (2014). Isolation, identification and characterization of lytic, wide host range bacteriophages from waste effluents against *Salmonella enterica* serovars. *Food Control*.

[57] Abatángelo V., Peressutti Bacci N., Boncompain C. A. (2017). Broad-range lytic bacteriophages that kill Staphylococcus aureus local field strains. *PLoS One*.

[58] Jurczak-Kurek A., Gąsior T., Nejman-Faleńczyk B. (2016). Biodiversity of bacteriophages: morphological and biological properties of a large group of phages isolated from urban sewage. *Scientific Reports*.

[59] Chen Y., Sun E., Song J., Yang L., Wu B. (2018). Complete genome sequence of a novel T7-like bacteriophage from a Pasteurella multocida capsular type a isolate. *Current Microbiology*.

[60] Brouwer M. S. M., Roberts A. P., Hussain H., Williams R. J., Allan E., Mullany P. (2013). Horizontal gene transfer converts non-toxigenic *Clostridium difficile* strains into toxin producers. *Nature Communications*.

[61] Sampson Goss M. A. (2020). Statistical analysis in JASP a guide for students. JASP v 0.14. https://jasp-stats.org/wp-content/uploads/2020/11/Statistical-Analysis-in-JASP-A-Students-Guide-v14-Nov2020.pdf.

